# Assessment of Diazotrophic *Proteobacteria* in Sugarcane Rhizosphere When Intercropped With Legumes (Peanut and Soybean) in the Field

**DOI:** 10.3389/fmicb.2020.01814

**Published:** 2020-07-31

**Authors:** Manoj Kumar Solanki, Zhen Wang, Fei-Yong Wang, Chang-Ning Li, Chhedi Lal Gupta, Rajesh Kumar Singh, Mukesh Kumar Malviya, Pratiksha Singh, Li-Tao Yang, Yang-Rui Li

**Affiliations:** ^1^Guangxi Crop Genetic Improvement and Biotechnology Lab, Guangxi Academy of Agricultural Sciences, Nanning, China; ^2^Department of Food Quality & Safety, The Volcani Center, Institute for Post-harvest and Food Sciences, Agricultural Research Organization, Rishon LeZion, Israel; ^3^College of Biology and Pharmacy, Yulin Normal University, Yulin, China; ^4^State Key Laboratory for Conservation and Utilization of Subtropical Agro-bioresources, Agricultural College, Guangxi University, Nanning, China; ^5^Guangxi Key Laboratory of Sugarcane Genetic Improvement, Key Laboratory of Sugarcane Biotechnology and Genetic Improvement (Guangxi), Ministry of Agriculture, Sugarcane Research Institute of Guangxi Academy of Agricultural Sciences, Sugarcane Research Center of Chinese Academy of Agricultural Sciences, Nanning, China; ^6^The Volcani Center, Institute of Soil, Water and Environmental Sciences, Agricultural Research Organization, Rishon LeZion, Israel

**Keywords:** intercropping, microbial community, high throughput sequencing, NifH gene, sugarcane

## Abstract

Several factors influenced the sugarcane production, and among them, higher use of nitrogen and depletion of soil nutrient constitutes a significant concern in China. Sugarcane-legume intercropping may help to regulate the microbial structure and functions. In the present study, sugarcane rhizosphere soils of three cropping systems: Sugarcane only (S-only), sugarcane with peanut (S + P), and sugarcane + soybean (S + S) were sampled in tillering, elongation, and maturation stages from two different experimental fields. High-throughput sequencing technologies applied to assess the effects of different cropping systems on the structure of nitrogenase (*nifH*) gene communities. A total of 3818 OTUs (operational taxonomic units) were acquired from all soil samples. Intercropping systems noticeably increased the relative abundance of *Proteobacteria* in the tillering stage. The increased microbial diversity in the rhizosphere was mainly due to soil organic carbon and total soil N. In contrast, intercropping has no significant negative impact on microbial abundance, but sugarcane growth stages influence it significantly, and two bacteria (*Bradyrhizobium* and *Pseudacidovorax*) showed significant shift during plant growth. The results provide insight into the microbial structure of *Proteobacteria* in the sugarcane legume-intercropping field, and how microbial community behaves in different growth stages. It can boost the microbial activity of the soil, and that could be a new strategy to stimulate soil fertility without causing any negative impact on crop production.

## Introduction

Globally, sugarcane is a leading source of sugar and biofuel. In China, where tropical and subtropical summer rainfall climate predominates, sugarcane has emerged as an excellent substitute in agriculture, because it can grow well during the dry season. In the past few years, Guangxi province occupied an essential place in the Chinese sugar industry (Li and Yang, 2015). Still, sugar production suffers from abiotic or biotic factors every year in China (Deng et al., 2017). Nutrient depletion in the soil is the major abiotic factor in Guangxi, and to get higher sugarcane production, balanced use of nitrogen fertilizer is the crucial factor (Thorburn et al., 2017). Biological nitrogen fixation (BNF) approved as a long-term solution that can fix the nitrogen without any negative impact on the environment (Vitousek et al., 2002; Iannetta et al., 2016). Soil and rhizosphere associated diazotrophs (N-fixers) are well-known for their contributions in N mineralization and cycling (Herridge et al., 2008; Hsu and Buckley, 2008; Li et al., 2016b; Zhang et al., 2017; Gupta et al., 2019). Among all soil nutrients, nitrogen is essential for plant growth and development, and plant assimilates the nitrogen from the soil as nitrite, nitrate, or ammonia (Horel et al., 2019). The significant phyla of diazotrophs bacteria are *Actinobacteria, Bacteroidetes, Cyanobacteria, Chlorobi, Chloroflexi, Firmicutes*, and *Proteobacteria* (Ganzert et al., 2014; Pérez-Montaño et al., 2014; Szymańska et al., 2018). Among all phyla, *Proteobacteria* significantly associated with the plant rhizosphere, and several non-symbiotic *Proteobacteria* have been acknowledged as free-living diazotrophs such as *Azohydromonas, Azospirillum, Azospira, Azoarcus, Azotobacter, Burkholderia, Herbaspirillum, Pelomonas, Pseudacidovorax*, and *Sphingomonas* (Chen et al., 2003; Aoki et al., 2013; Pankievicz et al., 2015; Roley et al., 2019).

Sugarcane cropping with other crops has taken worldwide attention to managing soil health and plant productivity (Singh et al., 2010; Dai et al., 2013; Li et al., 2013, 2018). China and sub-Saharan Africa have discovered better yielding and nutrient acquisition benefits under adverse conditions when cereal has grown with the legumes (Zhang and Li, 2003; Kermah et al., 2017; Solanki et al., 2017). Plant root-associated microbes are involved in the symbiosis of nutrients with other microbes and plants (Rilling et al., 2018). Several researchers reported diazotrophic soil bacteria as plant growth promoters of sugarcane that can associate with legumes as well as other crops (Chen et al., 2001, 2005; Garau et al., 2009; Bontemps et al., 2010; Castro-González et al., 2011; Paungfoo-Lonhienne et al., 2016). These are the most efficient and harmless sources for soil nourishment and increase agricultural production. Microbial characterization of plant rhizosphere is essential to understand the role of soil diazotrophs in N assimilation. So far, culture-independent methods have investigated for N fixation in different habitats, including soils (Zehr et al., 2003; Izquierdo and Nüsslein, 2006; Chowdhury et al., 2009; Li et al., 2012; Solanki et al., 2019b), plant parts (Lovell et al., 2001; Chowdhury et al., 2009) and water resources (Blais et al., 2012; Tai et al., 2013).

High throughput sequencing (HTS) generates more information than Sanger sequencing (Collavino et al., 2014; Gaby et al., 2018). Therefore, to acquire more data about rhizosphere associated diazotrophs, researchers are using HTS to unlock the complex microbial structure (Caporaso et al., 2012; Rascovan et al., 2016; Zhang et al., 2017). The *nifH* gene, which encodes a subunit of the nitrogenase enzyme, offers a convenient marker and that used to determine the distribution and diversity of diazotrophs in diverse environments (Coelho et al., 2009; Zou et al., 2011; Collavino et al., 2014). Investigation of *nifH* diversity in soil and rhizosphere, commonly disclose unidentified diazotrophs sequences (Poly et al., 2001; Buckley et al., 2007; Gaby et al., 2018). Past research evidence directs that these non-cultivated diazotrophs are dominant organisms in different soil systems as compared to cultivated diazotrophs (Hsu and Buckley, 2008), and sugarcane rhizosphere-biome in regards to the diazotrophs remain mostly obscure during intercropping with the legume. Therefore, we characterized the sugarcane rhizosphere diazotrophs during plant development when peanut and soybean crops were used as intercrop in the field. By sampling of rhizosphere soil of two different experimental areas in three different stages such as tillering, elongation and maturation, and analysis of the microbial composition, distribution, and dynamics of diazotrophs in a commercial sugarcane variety and their correlation with the soil parameters might help to understand the microbial structure in sugarcane rhizosphere.

## Materials and Methods

### Plant Material, Field Plan, and Sampling

Sugarcane (*var* GT31), peanut (*var* GH771), and soybean (*var* GC8) were obtained by the breeding unit of Sugarcane Research Institute and Cash Crop Research Institute, Guangxi Academy of Agricultural Sciences (GXAAS), Nanning, Guangxi, China. Two field experiments were carried out during the spring season at the experimental field station of Sugarcane Research Institute, GXAAS/SRC, CAAS, Nanning, Guangxi, China. Red loamy lateritic red earth (lato sol) used in this study. The details of soil and weather have been given in Supplementary Table S1. Three treatments were used with three replications: sugarcane only (S-only), sugarcane with peanut (S + P), and sugarcane with soybean (S + S) (Supplementary Figures S1, S2). Manual plantation of all three crops was performed into the soil during March-April 2014 in both experimental fields. Soil samples were accomplished at three growth stages of sugarcane; tillering, elongation, and maturation, respectively. Twenty rhizospheric soil (tightly adhering soil of root) samples were collected with a brush within 2 mm of the sugarcane root surfaces at each growth stage, passed through a 2 mm sieve, and stored at −20°C for analysis. Cane height and yield were measured manually at the end of the experiment.

### DNA Extractions and *nifH* Gene PCR Amplification

Genomic DNA was extracted from soil samples by using GnS-GII protocol (Plassart et al., 2012) and purified by the Ezup Column Soil DNA Purification Kit (Sangon Biotech, Shanghai, China). DNA quality and quantity were detected by NanoDrop ND-2000 UV-Vis Spectrophotometer (Thermo Fisher Scientific, Wilmington, DE, United States). Degenerate Z-primers (Zehr et al., 1998) were used to amplify the nitrogenase (*nifH*) gene through a Nested polymerase chain reaction (PCR) in the Peltier Thermal Cycler (Bio-Rad, Hercules, United States). Each 25 μl reaction contained 12.5 μl ready to use PCR mix (Tiangen Biotech, Beijing, China), 1.0 μl of each primer (10 μM), 2.5 μl of DNA template (10 ng/ml), and 9.0 μl PCR grade water. Outer primers (*nifH*3 and *nifH*4) were used for the first PCR (94°C-4 min, 30 cycles of 1 min at 94°C, 55°C, and 72°C, final extension at 72°C for 7 min), and inner primers (*nifH*1 and *nifH*2) were used with first PCR-product as a template followed by a touchdown PCR strategy. First, 20 touchdown cycles were performed by a reduction of 0.5°C per cycle ranging from 67 to 57°C, and rest 15 cycles were performed with the annealing temperature of 57°C. Purification of PCR products was done by TIANgel Midi Purification Kit (Tiangen Biotech, Beijing, China). T_4_ DNA polymerase, Klenow Fragment, and T_4_ Polynucleotide Kinase were used to change jagged ends in to blunt ends. Then sequencing adapters were added to each end of amplicons to construct libraries and qualified library was used for high-throughput sequencing with the Illumina Miseq sequencer platform.

### Bioinformatics

Illumina generated paired-end sequences were processed using the QIIME 2 v2018.11 bioinformatics pipeline (Bolyen et al., 2019). The obtained sequences were initially undergone for quality filtering employing DADA2 algorithm (Callahan et al., 2016) that resolves amplicon-sequencing errors to generate amplicon sequence variants (ASVs). Moreover, we used an analysis pipeline named TaxADivA, which uses their own well-curated *nifH* gene database for diazotroph community characterization in high-throughput *nifH* amplicon sequencing (Gaby et al., 2018). Hence, we used this custom *nifH* gene database to train aQIIME Naïve Bayes classifier for taxonomic assignment of our sequences using Qiime feature-classifier option. Beta diversity heatmap and principal component analysis were performed by the software R (v3.0.3) in the QIIME pipeline. The taxonomic rank (Phylum to Species) and the histogram was drawn with the software R (v3.0.3). Circos plots were drawn by Circos Table Viewer v0.63-9 software (Krzywinski et al., 2009). Heatmaps and Venn plots were generated using the package “ggplots” of software R (v3.0.3). The Illumina generated sequence data was deposited to the National Center for Biotechnology Information (NCBI) under Bio-Project accession number: PRJNA310619 (Supplementary Table S3).

### Statistical Analysis

The experiments were conducted in replicates, and data were analyzed using standard analysis of variance (ANOVA) followed by the Tukey’s HSD tests all pairwise by Origin 2017SR2 software (Northampton, MA, United States). Soil chemical parameters and enzymes data were used from our previous study (Solanki et al., 2019a), to calculate Spearman’s rank correlation coefficient between soil variables and bacterial taxa by using PAST3 software (Hammer et al., 2001) and heatmap generates by using ClustVis online tool (Metsalu and Vilo, 2015).

## Results and Discussion

### Sequencing Results and Microbial Diversity

**FIGURE 1:**
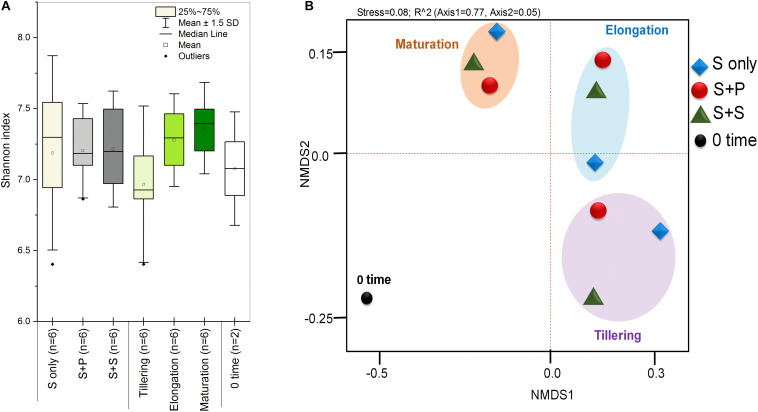
Box plots of Shannan diversity index **(A)**, Principal coordinate analysis (PCoA) based on the Bray-Curtis distance metrics of diazotrophs between the cropping systems and growth stages **(B)**. S only, Sugarcane monoculture; S + P, Sugarcane and Peanut intercropping; S + S, Sugarcane and Soybean intercropping.

**FIGURE 2:**
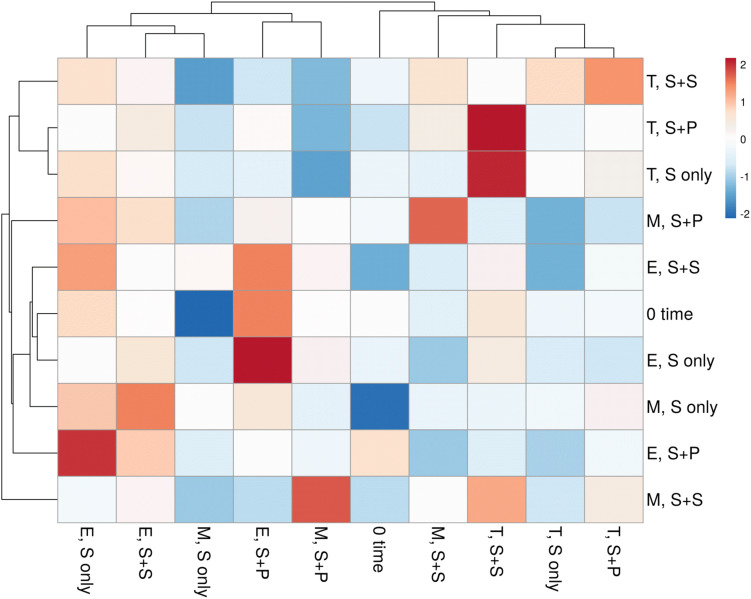
The heatmap of correlation between three cropping systems and three growth stages of sugarcane-based on OTUs profile. Red means a positive correlation, while blue represents a negative correlation. S only, Sugarcane monoculture; S + P, Sugarcane and Peanut intercropping; S + S-Sugarcane and Soybean intercropping.

HTS enlightens a modern approach to discover and classify the natural microbial niches, in a short time of period (Gaby and Buckley, 2014; Gaby et al., 2018). These tools are also utilized for different kinds of environmental samples (Izquierdo and Nüsslein, 2006; Chowdhury et al., 2009; Li et al., 2012). Sugarcane is a long time perennial grass crop, and root-associated microbes play an essential role in each growth stage. To understand the functional diversity, composition, structure, and dynamics of rhizospheric diazotrophs communities under different cropping systems, we isolated the soil DNA and amplified a ∼360bp *nifH* gene fragment by nested PCR. The utility of the nested PCR method has been well-established in earlier studies of *nifH* gene diversity (Jenkins et al., 2004; Orr et al., 2011; Blais et al., 2012; Liu et al., 2012). A total of 812,292 sequences were obtained through the High throughput sequencing of 20 soil samples, and after quality filtration, a total of 786,283 sequences were found. A total of 644,145, high-quality paired-end reads were used to remove Chimeras, and a total of 801,126 non-chimeric sequences were obtained in a total average of 13,263 sequences per sample, and the average length is 358–366 bp with ~99% connecting ratio. Non-chimeric sequences were clustered into Operational Taxonomic Units (OTU) at 97% similarity, and a total of 3818OTUs were acquired. No statistically significant difference was detected between cropping systems and sugarcane growth stages based on the Shannon index, but the S + S intercrop and maturation stage showed higher Shannon index values (7.22 ± 0.11 and 7.36 ± 0.09), respectively (Figure 1A). The Shannon diversity index of the *nifH* gene ranged 6.40–7.64 in this study, which is higher than those in other studies (Coelho et al., 2009; Jungblut and Neilan, 2010; Niederberger et al., 2012; Tai et al., 2013,2014; Zhang et al., 2017). These results concluded that the monoculture cultivation system might alter the ecological environment of soil microorganisms, and thereby causing reductions of bacterial communities in the soil. On the other hand, Non-metric multidimensional scaling (NMDS) analysis based on the Bray Curtis dissimilarity metric, all three cropping systems (S-only, S + P, and S + S) varied in tillering and elongation (Figure 1B). Similarly, a beta-diversity heat map and UPGMA clustering based on Bray Curtis dissimilarity metric showed robust clustering among cropping systems and growth stages. In the heatmap, column-wise, and raw-wise, all three cropping systems clustered together in the tillering (Figure 2). Next, treatment S-only of elongation and maturation stages was gathered together, S + P and S + S also grouped in elongation and maturation stage, respectively (Figure 2). Solanki et al. (2019b) reported similar kinds of results by the survey of intercropped fields of formers from different locations of Nanning. However, in the study of Solanki et al. (2019b), less microbial taxa identified from sugarcane rhizosphere due to use of the Green genes database for identification of the *nifH* gene community. In the present study, we used the *nifH* gene database pipeline TaxADivA, which is specially designed by Gaby et al. (2018) to analyze the *nifH* gene community. The outcome of the present study is also consistent with several researchers who verified that grass-legume intercropping enhanced the microbial diversity of soil (Li et al., 2013, 2016c; Lian et al., 2019). However, a dramatic change in the Shannon index, and beta diversity NMDS plot, also reflecting on the nutritional depletion of soil under the elongation and maturation stages. Dong-Hai et al. (2014) reported that sugarcane-soybean intercropping had significant effects on the diversity of nitrogen-fixing bacteria in the rhizosphere of sugarcane. Recently, Zhou et al. (2017) demonstrated that legumes crops improve the soil microbial community higher than grass crops. In the present study, we found higher diversity in the intercropped sugarcane as compared to monoculture, and among intercropped, soybean showed higher diversity than peanut intercropping. It may be due to the root exudates of multiple plantscan boost the soil microbial taxa. The bacterial community in intercropping may contact the crop roots directly, and this interaction may stimulate the plant root to release exudates and nutrients (Haldar and Sengupta, 2015; Canarini et al., 2019).

### Microbial Distribution and Abundance

**FIGURE 3:**
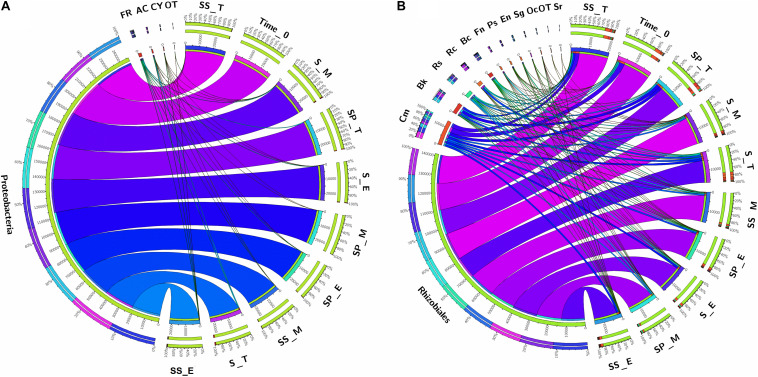
Circular representation of the proportional structure of bacterial communities at the phylum level **(A)** and Order level **(B)** associated with the sugarcane rhizosphere in different growth stages. Taxa with a proportion lower than 0.1% in all samples are summarized as “OT-Others.” Values within the inner circle indicate the number of reads of a phylum and Order within the normalized dataset. S, Sugarcane only; SP, Sugarcane + Peanut; SS, Sugarcane + Soybean; T, Tillering; E, Elongation; M, Maturation; FR, *Firmicutes*; AC, *Actinobacteria*; CY, *Cyanobacteria*; Cm, *Chromatiales*; Bk, *Burkholderiales*; Rs, *Rhodospirillales*; Rc, *Rhodocyclales*; Bc, *Bacillales*; Fn, *Frankiales*; Ps, *Pseudomonadales*; En, *Enterobacteriales*; Sg, *Sphingomonadales*; Oc, *Oscillatoriales*; Sr, *Spirochaetales*; OT, Others.

**FIGURE 4:**
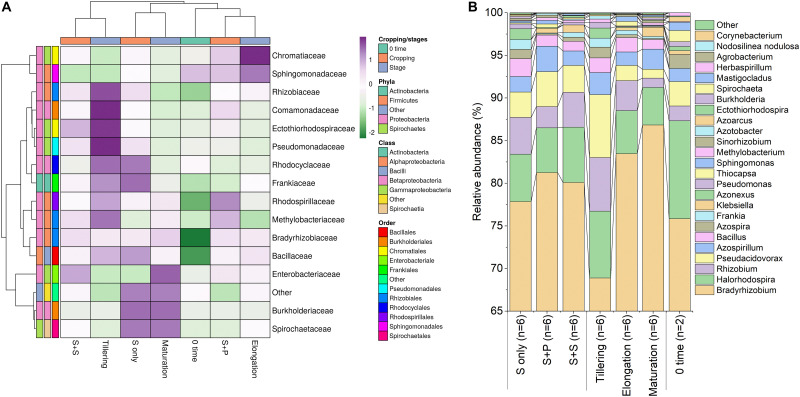
Relative abundance heatmap of diazotrophic bacterial taxa at the family level **(A)**, a Bar graph of relative abundance of diazotrophs at the genus level **(B)**. The most abundant classes are shown. Taxa with a proportion lower than 0.1% in all samples are summarized as “Others.” S only, Sugarcane monoculture; S + P, Sugarcane and Peanut intercropping; S + S, Sugarcane and Soybean intercropping.

**FIGURE 5:**
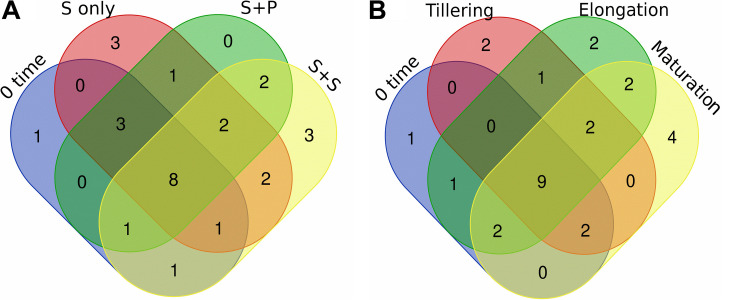
Venn diagram of diazotrophs at the genus level that distributed in different cropping systems **(A)** and growth stages **(B)**. Unique and shared OTUs between the sample pairs are based on 97% similarity. Taxa with a proportion lower than 0.1% not used in this analysis. S only, Sugarcane monoculture; S + P, Sugarcane and Peanut intercropping; S + S, Sugarcane and Soybean intercropping.

As, identification and taxonomic classification of organisms requires a reference database, which is usually available for universal genes such as 16S (the taxonomic marker gene for bacteria) (Maidak et al., 2001), a database with the same level of functionality has not been well-developed for the most functional genes including *nifH* gene. In the present study, we utilized TaxADivA pipeline to analyze *nifH* gene community (Gaby et al., 2018). The result shows that cropping systems have a significant impact on diazotrophic microbiome. Circos plot showing the relative abundance and microbial distribution among all the samples (Figure 3). The dominant phylum in the sugarcane rhizosphere of all samples turned out to be *Proteobacteria*, which accounted for 98–99% in all three cropping systems (Figure 3A). Other phyla identified in all soil samples were *Firmicutes*, *Actinobacteria*, and *Cyanobacteria*. The phylum *Actinobacteria* was higher in S + S treatment in elongation and maturation stages. When comparing the OTUs classification at class level, *Proteobacteria* phyla was divided into three sub-groups: *Alphaproteobacteria* (85%), *Gammaproteobacteria* (10%), and *Betaproteobacteria* (4%) (Supplementary Figure S3). The treatment S + P was showed higher OTU numbers in the case of class *Alphaproteobacteria* in the maturation stage, whereas S + S showed higher OTU numbers in the case of *Gammaproteobacteria* in the tillering, and the maximum OTUs of *Betaproteobacteria* were found in S-only treatment during tillering. In the case of *Bacilli* and *Actinobacteria*, the maximum OTUs resulted in S-only treatment in the tillering stage (Supplementary Figure S3). Differences in the effects of cropping systems were also noticeable at the order level (Figure 3B). OTU abundance in the order rank was affected to a greater extent by both intercropping systems. Three dominated order *Rhizobiales* (83.7% each), *Chromatiales* (6.4 and 7.9%), and *Burkholderiales* (4.1 and 2.7%) that covers 93% total abundance were found higher in S + P and S + S samples, respectively. However, Orders such as *Rhodocyclales*, *Bacillales*, *Frankiales*, and *Pseudomonadales* have an adverse effect by the intercropping systems. In the case of growth stages, except *Rhizobiales*, other dominated orders (*Chromatiales*, *Burkholderiales*, *Rhodospirillales*, *Rhodocyclales*, *Bacillales*, *Frankiales*, and *Pseudomonadales*) determined higher in tillering stage (Figure 3B). *Rhizobiales* order abundance was higher in the maturation stage. To get an overall view of the identified connections among the samples, hierarchically clustered heatmaps were generated (Figure 4A). The closer the color was to the purple, the more dominant microorganism was. There were differences among cropping systems and growth stages. According to the heatmaps, the fluctuation of bacterial communities in 0-time was lower than other treatments. Cropping system S + S and tillering stage were clustered together. Among the three cropping systems, the activity of bacteria was the lowest in S + P. However, the tillering stage samples showed dominant bacterial activity at the family level (Figure 4A). Moreover, differences in OTU abundance in the sugarcane rhizosphere were also calculated at the family level (Supplementary Figure S4). In the S-only treatment, the highest number of OTUs was determined for the families: *Bradyrhizobiaceae* (77.4%), *Ectothiorhodospiraceae* (5.7%), *Rhizobiaceae* (4.3%), Comamonadaceae (2.8%), *Rhodocyclaceae* (2.7%), *Bacillaceae* (2.1), and *Rhodospirillaceae* (1.8%). In the S + P treatment, the order of families acc. to OTUs number was as follows: *Bradyrhizobiaceae* (79.5%), *Ectothiorhodospiraceae* (5.3%), *Comamonadaceae* (4.6%), *Rhodospirillaceae* (3.2%), *Rhizobiaceae* (3.1%), and *Bacillaceae* (1.3%), whereas in the S + S treatment, it was: *Bradyrhizobiaceae* (78.9%), *Ectothiorhodospiraceae* (7.7%), *Rhizobiaceae* (3.9%), *Comamonadaceae* (3.1%), *Rhodospirillaceae* (1.8%), and *Bacillaceae* (1.1%). Except for family *Ectothiorhodospiraceae*, other family abundance identified higher in all three cropping systems as compared to the 0-time. In the case of stages, a higher number of OTUs found in tillering, followed by maturation and elongation (Supplementary Figure S4). At the genus level, 28 genera belonging to the 6 phyla were detected in the samples. In total, 25 most abundant shared genera with a relative abundance? ≥?0.01% were present in all samples across different groups, but their relative abundance levels were markedly different among the different cropping systems and growth stages (Figure 4B). The lower bacterial activity was detected in 0-time samples as compared to others. *Bradyrhizobium* (78.9%) was the most prevalent genus followed by *Halorhodospira* (6.8%), *Pseudacidovorax* (3.4%), *Rhizobium* (3.3%), *Azospirillum* (2.2%), *Bacillus* (1.3%), Azospira (0.7%), *Azonexus* (0.5%), *Frankia* (0.5%), *Klebsiella* (0.4%), *Pseudomonas* (0.3%), *Thiocapsa* (0.3%), *Sphingomonas* (0.3%), *Methylobacterium* (0.2%), and *Sinorhizobium* (0.2%) (Figure 3B). The *Rhizobium*, *Bacillus*, *Azospira*, *Frankia*, *Azonexus*, *Pseudomonas*, *Burkholderia*, and *Spirochaeta* made up the abundant bacterial genera in S-only treatment as compared to both intercropping treatments. The *Bradyrhizobiumi*, *Pseudacidovorax*, *Azospirillum*, *Thiocapsa*, *Sphingomonas*, *Methylobacterium*, and *Sinorhizobium* were the seven most abundant genera in the samples from the S + P treatment, while the *Halorhodospira* and *Klebsiella* were more prominent in the S + S treatment. The *Azoarcus* and *Herbaspirillum* were found abundant in 0-time soil samples (Figure 4B). Most of the dominant genera were found in the tillering stage, except *Bradrhizobiumi* and *Klebsiella.* They were dominant in the maturation stage (Figure 3B). Moreover, genus abundance-based Venn diagram showed that eight genera (*Azospirillum*, *Rhizobium*, *Bradyrhizobium*, *Halorhodospira*, *Pseudacidovorax*, *Methylobacterium*, *Desulfovibrio*, and *Azospira*) found common in all samples (Figure 5A). Genus *Sphingomonas*, *Thiocapsa*, and *Azonexus* commonly existed in 0 time, S only, and S + P samples. Genus *Frankia* and *Bacillus* were found common in all three cropping systems (S only, S + P, and S + S). Two genes *Azotobacter* and *Agrobacterium*, were found common in S + P and S + S samples. Although, genes *Corynebacterium*, *Pelodictyon*, *Burkholderia*were found only in S only samples. Three unique genera (*Actinobacteria*, *Azoarcus*, and *Mastigocladus*) were found in S + S samples (Figure 5A). Venn diagram based on sugarcane growth stages determined that nine genera such as *Azospirillum, Rhizobium, Bradyrhizobium, Halorhodospira, Pseudacidovorax, Azonexus, Desulfovibrio, Pseudomonas*, and *Azospira* were found common in all samples. Maximum unique genera (*Corynebacterium, Pelodictyon, Burkholderia*, and *Mastigocladus*) were found in the maturation stage (Figure 5B). Furthermore, we analyzed the individual genus by box plot. The genus *Bradyrhizobium* and *Pseudacidovorax* significantly (*P* < 0.05) influenced by sugarcane growth stages. *Bradyrhizobium* abundance significantly (*P* < 0.05) enlarged in the maturation stage. Besides, *Pseudacidovorax* abundance concentrated substantially at the maturation stage (Supplementary Figure S5). These results also matched with past studies executed by various molecular tools (Orr et al., 2011; Blais et al., 2012; Li et al., 2012; Yousuf et al., 2014; Solanki et al., 2019b). *Proteobacteria* is a relatively abundant phylum that is commonly found in sugarcane soil (Pisa et al., 2011; Solanki et al., 2019b). The high abundance of *Pseudacidovorax* was found in all cropping systems in the tillering stage and *Bradyrhizobium* in elongation and maturation, and these results showed that the functional shift of diazotrophs according to sugarcane growth. Several past reports also reported the association of *Bradyrhizobium* with the non-leguminous plants (Rouws et al., 2014a; Nyoki and Ndakidemi, 2018b; De Alencar et al., 2019; Hara et al., 2019; Wasai-Hara et al., 2020). Nyoki and Ndakidemi (2018b) described that inoculation of *Bradyrhizobium* with soybean and maize improves the crop health and yield significantly. Irrespective of diazotrophs, *alphaproteo bacteria* have commonly existed in the rhizosphere of several grass crops (Yousuf et al., 2014; Solanki et al., 2017). Likewise, *Bradyrhizobium* sp. is more competent to colonize the roots of non-leguminous plants like sugarcane (Rouws et al., 2014b; Solanki et al., 2019b). *Pseudacidovorax genus* has been already perceived as active diazotrophs in soil, plant, and water (Zhang and Chen, 2012; Fu and Zheng, 2016; Wedage et al., 2019). Besides, two genera play diverse functions in tillering, elongation, and maturation stages, concluded that different kinds of soil biota have distinct types of actions in soil nutrient mineralization, and they directly influenced by the plant root exudates (Canarini et al., 2019).

### Correlation Analysis

**FIGURE 6:**
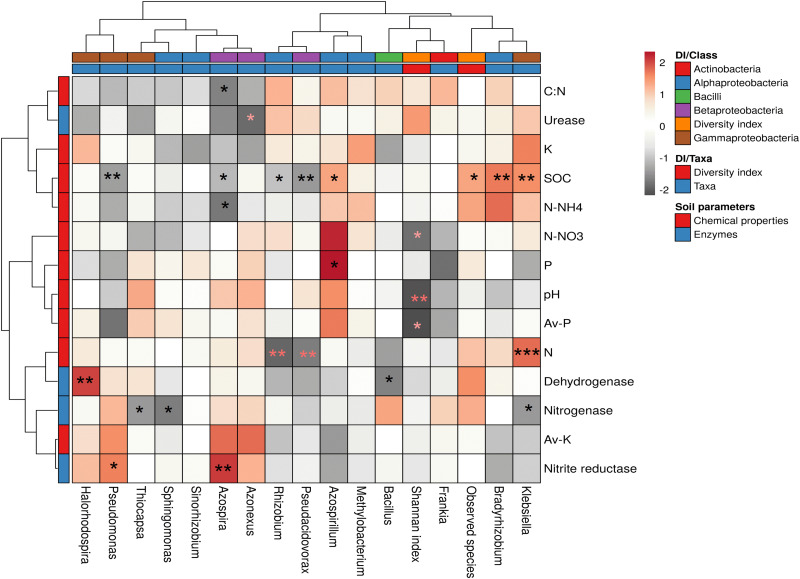
Spearman’s rank correlation between the soil parameters and microbial community of sugarcane rhizosphere. *P < 0.1, **P < 0.05, ***P < 0.01. Av, available; DI, diversity index; SOC, soil organic carbon.

Plant root, rhizosphere, and non-rhizospheric soil microflora are playing significant roles in plant growth promotion and soil mineralization. Various findings reported that soil microflora, soil enzyme activities, and crop production might be influenced by different land management practices (Zou et al., 2011; Yang et al., 2013; Wang et al., 2014). However, in the present study, we found that the intercropping system did not cause any negative impact on cane growth and yield (Supplementary Figure S6). Hauggaard-Nielsen et al. (2012) and Zaeem et al. (2019) reported that intercropping crops boosts the soil nutrients. These reports help us to infer that intercropping promote direct and indirect benefits for sugarcane plants which may be associated with actions of different soil microbes to solubilize minerals and protect the plant from pathogens. Mineral solubilization is an important method of soil microbes in the intercropped crops (Zhang and Li, 2003; Wang et al., 2014; Iannetta et al., 2016; Li et al., 2016a). In the present study, the Spearman’s rank correlation analysis was calculated among all the chemical properties, and taxon abundance at genus levels and diversity index for diazotrophs, and values were illuminated in a heat map (Figure 6). Highly significant correlations were observed between various soil properties, enzyme activities, and diazotrophs community. A highly significant positive correlation was observed between chemical properties, i.e., SOC and total N, P, and bacterial taxa, i.e., *Klebsiella* and *Azospirillum.* A negative correlation between soil pH and Shannan index concluded that low pH reduced microbial diversity. However, the abundance of genus *Azospirillum*, *Thiocapsa*, and *Azonexus* show a positive correlation with soil pH as compared to other diazotrophs, and these bacteria probably help the sugarcane plant to reduce soil acidity. Our results collaborated with past reports of Nyoki and Ndakidemi (2018a), who found that diazotrophic bacteria such as *Rhizobium* reduced the soil acidity in the rhizosphere of soybean and increased the soil pH, which favored the availability of plant nutrients (Bagayoko et al., 2000; Nyoki and Ndakidemi, 2018a). A high positive correlation between soil organic carbon (SOC) and abundance of bacteria such as *Bradyrhizobium* (*r* = 0.50, *P* < 0.05), *Klebsiella* (*r* = 0.46, *P* < 0.05), and *Azospirillum* (*r* = 0.40, *P* < 0.1) revealed the microbial association with C cycling. Norman and Friesen (2017) reported that BNF is a complex process of diazotrophs that needs a higher amount of organic C. Moreover, a significantly negative correlation was observed between SOC and abundance of the genus such as *Pseudacidovorax, Pseudomonas, Rhizobium*, and *Azospira* (Figure 6). Rhizospheric soil is a complex system, and higher amount of soil carbon increased the diffusive transport of organic soluble substrates in diazotrophs that enhance the microbial mobility in the rhizosphere (Ding et al., 2015; Chen et al., 2019). Intercropping improves the organics matter of soil through a higher microbial activity that also influences the soil pH (Solanki et al., 2017; Layek et al., 2018). For soil minerals, a significant positive link found between total N and abundance of genus *Klebsiella* (*r* = 0.56, *P* < 0.01), and a negative link with *Rhizobium* and *Pseudacidovorax* (Figure 6). These results directed that microbial diversity influenced the soil N. A positive correlation between soil C:N ratio and abundance of bacteria such as *Rhizobium, Frankia, Azospirillum*, and *Bradyrhizobium* represent the importance of diazotrophs in carbon and nitrogen cycles. However, few less abundant bacteria such as *Azospira*, *Azonexus*, and *Pseudomonas* showed a negative correlation with C:N ratio. Moreover, abundance of genus *Bradyrhizobium* and *Azospirillum* showed a positive association with available N–NH_4_ and N- NO_3_^–^, respectively. Although, a robust negative association between available N like N–NH_4_ and N- NO_3_^–^, and abundance of genus *Azospira* (Figure 6), and Shannon diversity indicated that different diazotrophs genera had the differential kind of functions in the sugarcane rhizosphere. A positive correlation between total and available P, and abundance of genus *Azospirillum* revealed the importance of mineral solubilization in the sugarcane rhizosphere. These results collaborated with past reports, who reported that soil P played a significant role in microbial growth and plant development (Bagayoko et al., 2000; Ding et al., 2015). Additionally, a positive correlation between available K and abundance of *Azospira* and *Azonexus* revealed that few bacteria plays important role in K mineralization to balance soil nutrients. Thomas and Hungria (1988) reported that K played a significant role in nitrogenase activity. In the present study, positive correlation between total K and abundance of genus *Klebsiella* and *Methylobacterium* recommend that these microbes immobilize the soil K and transport it to the plant. Soil N played a significant role in sugarcane tillering (Leite et al., 2016), although soil K also plays a crucial role in the photosynthesis under stressed conditions (Shukla et al., 2009). In the case of soil enzyme nitrite reductase, a significant positive links resulted with the abundance of genus *Azospira*. On the contrary, a negative correlation between nitrogenase enzyme and abundance of genus *Sphingomonas*, *Thiocapsa*, and *Klebsiella* concluded that soil nitrogen content and other microbes influenced the enzyme production. Furthermore, a significant positive correlation between enzyme dehydrogenase and abundance of *Halorhodospira* revealed that few microbes maintained microbial activity even in nutrient depletion condition. On the contrary, soil enzymes such as urease and dehydrogenase showed a negative correlation with abundance of genus *Azonexus* and *Bacillus*, respectively, and it may be due to reduction of soil nutrients in sugarcane rhizosphere that enhance the microbial completion (Zong et al., 2015; Jones et al., 2018). Intercropping improved the organic matter in soil that influenced the microbial activity and plant growth (Verma et al., 2014; Duchene et al., 2017). The correlation analysis also signifies that soil biochemical properties correlated with the different kind of bacterial genus. Differential patterns of microbial niches also associated with environmental factors, and sugarcane root and soil association.

## Conclusion

In conclusion, the intercropping system enriched the soil organic carbon that helps the diazotrophs to propagate at the tillering stage. These diazotrophs immobilize the soil nutrients that help sugarcane plant to stimulate their growth. A long-duration crop interacts with diverse kinds of taxa to cope with their requirement, and when it has grown with short duration crops during tillering, soil nutrients competition increased, and it reduced in elongation and maturation. However, intercropping crops helps to utilize the maximum amount of soil nutrients through diverse kinds to microbes, and directly or indirectly, this process reduces the growth of other microbes. High throughput sequencing results of *nifH* gene provide information in-depth about diazotrophic *Proteobacteria*, and higher abundance of *Bradyrhizobium* played a significant role in sugarcane growth. Soil organic carbon, nitrogen and nitrite reductase enzyme had a significant correlation with microbial diversity. The present study represents an insight into the HTS technology application under different cropping systems as well as different growth stages. Hence, further investigation is needed to utilize intercropping to boost soil BNFs and PGPRs in sugarcane rhizosphere. More attention that is considered must be paid to studies and application of new combinations of intercropping with legumes and short duration vegetables with a long duration crop like sugarcane that can help to balance the soil nutrients and utilize the space in an efficient manner.

## Data Availability Statement

The datasets generated for this study can be found in the NCBI under accession number PRJNA310619.

## Author Contributions

MS, Y-RL, and L-TY conceived and designed the experiments and wrote the manuscript. MS, F-YW, ZW, and C-NL performed the experiments. MS, C-NL, ZW, and CG analyzed the data. MM, CG, RS, and PS contributed to the reagents, materials, and analysis tools. All authors contributed to the article and approved the submitted version.

## Conflict of Interest

The authors declare that the research was conducted in the absence of any commercial or financial relationships that could be construed as a potential conflict of interest.

## References

[B1] Aoki S. Ito M. Iwasaki W. (2013). From β- to α-*Proteobacteria*: the origin and evolution of rhizobial nodulation genes nodIJ. *Mol. Biol. Evol.* 30 2494–2508. 10.1093/molbev/mst153 24030554

[B2] Bagayoko M. Alvey S. Neumann G. Buerkert A. (2000). Root-induced increases in soil pH and nutrient availability to field-grown cereals and legumes on acid sandy soils of Sudano-Sahelian West Africa. *Plant Soil* 225 117–127. 10.1023/A:1026570406777

[B3] Blais M. Tremblay J. É Jungblut A. D. Gagnon J. Martin J. Thaler M. (2012). Nitrogen fixation and identification of potential diazotrophs in the Canadian Arctic. *Global Biogeochem. Cycles* 26:GB3022 10.1029/2011GB004096

[B4] Bolyen E. Rideout J. R. Dillon M. R. Bokulich N. A. Abnet C. C. Al-Ghalith G. A. (2019). Reproducible, interactive, scalable and extensible microbiome data science using QIIME 2. *Nat. Biotechnol.* 37 852–857. 10.1038/s41587-019-0209-9 31341288 PMC7015180

[B5] Bontemps C. Elliott G. Simon M. Dos Reis F. Jr. Gross E. Lawton R. (2010). Burkholderia species are ancient symbionts of legumes. *Mol. Ecol.* 19 44–52. 10.1111/mec.2009.19.issue-1 20002602

[B6] Buckley D. H. Huangyutitham V. Hsu S.-F. Nelson T. A. (2007). Stable isotope probing with 15N2 reveals novel noncultivated diazotrophs in soil. *Appl. Environ. Microbiol.* 73 3196–3204. 10.1128/AEM.02610-06 17369332 PMC1907113

[B7] Callahan B. J. McMurdie P. J. Rosen M. J. Han A. W. Johnson A. J. A. Holmes S. P. (2016). DADA2: High-resolution sample inference from Illumina amplicon data. *Nat. Methods* 13 581–583. 10.1038/nmeth.3869 27214047 PMC4927377

[B8] Canarini A. Kaiser C. Merchant A. Richter A. Wanek W. (2019). Root exudation of primary metabolites: Mechanisms and their roles in plant responses to environmental stimuli. *Front. Plant Sci.* 10:157. 10.3389/fpls.2019.00157 30881364 PMC6407669

[B9] Caporaso J. G. Lauber C. L. Walters W. A. Berg-Lyons D. Huntley J. Fierer N. (2012). Ultra-high-throughput microbial community analysis on the Illumina HiSeq and MiSeq platforms. *ISME J.* 6 1621–1624. 10.1038/ismej.2012.8 22402401 PMC3400413

[B10] Castro-González R. Martínez-Aguilar L. Ramírez-Trujillo A. Estrada-de los Santos P. Caballero-Mellado J. (2011). High diversity of culturable Burkholderia species associated with sugarcane. *Plant Soil* 345 155–169. 10.1007/s11104-011-0768-0

[B11] Chen J. Shen W. Xu H. Li Y. Luo T. (2019). The composition of nitrogen-fixing microorganisms correlates with soil nitrogen content during reforestation: a comparison between legume and non-legume plantations. *Front. Microbiol.* 10:508. 10.3389/fmicb.2019.00508 30930882 PMC6427063

[B12] Chen W. M. De Faria S. M. Straliotto R. Pitard R. M. Simões-Araùjo J. L. Chou J. H. (2005). Proof that Burkholderia strains form effective symbioses with legumes: a study of novel Mimosa-nodulating strains from South America. *Appl. Environ. Microbiol.* 71 7461–7471. 10.1128/AEM.71.11.7461-7471.2005 16269788 PMC1287612

[B13] Chen W. M. Laevens S. Lee T. M. Coenye T. De Vos P. Mergeay M. (2001). Ralstonia taiwanensis sp. nov., isolated from root nodules of Mimosa species and sputum of a cystic fibrosis patient. *Int. J. Syst. Evol. Microbiol.* 51 1729–1735. 10.1099/00207713-51-5-1729 11594603

[B14] Chen W. M. Moulin L. Bontemps C. Vandamme P. Béna G. Boivin-Masson C. (2003). Legume symbiotic nitrogen fixation by β-*Proteobacteria* is widespread in nature. *J. Bacteriol.* 185 7266–7272. 10.1128/JB.185.24.7266-7272.2003 14645288 PMC296247

[B15] Chowdhury S. P. Schmid M. Tripathi A. K. (2009). Diversity of 16S-rRNA and nifH genes derived from rhizosphere soil and roots of an endemic drought tolerant grass, Lasiurus sindicus. *Eur. J. Soil Biol.* 45 114–122. 10.1016/J.EJSOBI.2008.06.005

[B16] Coelho M. R. R. Marriel I. E. Jenkins S. N. Lanyon C. V. Seldin L. O’Donnell A. G. (2009). Molecular detection and quantification of nifH gene sequences in the rhizosphere of sorghum (Sorghum bicolor) sown with two levels of nitrogen fertilizer. *Appl. Soil Ecol.* 42 48–53. 10.1016/J.APSOIL.2009.01.010

[B17] Collavino M. M. Tripp H. J. Frank I. E. Vidoz M. L. Calderoli P. A. Donato M. (2014). *nifH* pyrosequencing reveals the potential for location-specific soil chemistry to influence N_2_ -fixing community dynamics. *Environ. Microbiol.* 16 3211–3223. 10.1111/1462-2920.12423 25314671

[B18] Dai C.-C. Chen Y. Wang X.-X. Li P.-D. (2013). Effects of intercropping of peanut with the medicinal plant atractylodes lancea on soil microecology and peanut yield in subtropical China. *Agrofor. Syst.* 87 417–426. 10.1007/s10457-012-9563-z

[B19] De Alencar M. I. Jr. Feitosa De Matos G. Moura De Freitas K. Da Conceição Jesus E. Rouws L. F. M. (2019). Occurrence of diverse *Bradyrhizobium* spp. in roots and rhizospheres of two commercial Brazilian sugarcane cultivars. *Brazilian J. Microbiol.* 50 759–767. 10.1007/s42770-019-00090-6 31144269 PMC6863198

[B20] Deng Y. Han S. Huang Y. Yang Y. Huang Y. Wang L. (2017). An investigation report on production conditions of sugarcane in Guangxi in 2016. *Asian Agric. Res.* 9 79–83.

[B21] Ding K. Zhong L. Xin X. P. Xu Z. H. Kang X. M. Liu W. J. (2015). Effect of grazing on the abundance of functional genes associated with N cycling in three types of grassland in Inner Mongolia. *J. Soils Sediments* 15 683–693. 10.1007/s11368-014-1016-z

[B22] Dong-Hai P. Jian-Bo Y. Jian L. Yong-Xiu Xi Liu-Dong Q. Li-Tao Y. (2014). Effects of intercropping with soybean on bacterial and nitrogen-fixing bacterial diversity in the rhizosphere of sugarcane. *Chinese J. Plant Ecol.* 38 959–969. 10.3724/sp.j.1258.2014.00090

[B23] Duchene O. Vian J. F. Celette F. (2017). Intercropping with legume for agroecological cropping systems: Complementarity and facilitation processes and the importance of soil microorganisms: a review. *Agric. Ecosyst. Environ.* 240 148–161. 10.1016/j.agee.2017.02.019

[B24] Fu M. H. Zheng L. J. (2016). Effects of different forms of nitrogen on rhizosphere microbial community structure of Eichhornia crassipes (Pontederiaceae). *Rev. Biol. Trop.* 64 213–220. 10.15517/rbt.v64i1.18138 28862420

[B25] Gaby J. C. Buckley D. H. (2014). A comprehensive aligned nifH gene database: a multipurpose tool for studies of nitrogen-fixing bacteria. *Database* 2014:bau001. 10.1093/database/bau001 24501396 PMC3915025

[B26] Gaby J. C. Rishishwar L. Valderrama-Aguirre L. C. Green S. J. Valderrama-Aguirre A. Jordan I. K. (2018). Diazotroph community characterization via a highthroughput nifH amplicon sequencing and analysis pipeline. *Appl. Environ. Microbiol.* 84:e01512-17. 10.1128/AEM.01512-17 29180374 PMC5795091

[B27] Ganzert L. Bajerski F. Wagner D. (2014). Bacterial community composition and diversity of five different permafrost-affected soils of Northeast Greenland. *FEMS Microbiol. Ecol.* 89 426–441. 10.1111/1574-6941.12352 24819653

[B28] Garau G. Yates R. J. Deiana P. Howieson J. G. (2009). Novel strains of nodulating Burkholderia have a role in nitrogen fixation with papilionoid herbaceous legumes adapted to acid, infertile soils. *Soil Biol. Biochem.* 41 125–134. 10.1016/j.soilbio.2008.10.011

[B29] Gupta V. V. S. R. Zhang B. Penton C. R. Yu J. Tiedje J. M. (2019). Diazotroph diversity and nitrogen fixation in summer active perennial grasses in a mediterranean region agricultural soil. *Front. Mol. Biosci.* 6:115. 10.3389/fmolb.2019.00115 31750314 PMC6848460

[B30] Haldar S. Sengupta S. (2015). Plant-microbe cross-talk in the rhizosphere: insight and biotechnological potential. *Open Microbiol. J.* 9 1–7. 10.2174/1874285801509010001 25926899 PMC4406998

[B31] Hammer Ø Harper D. A. T. Ryan P. D. (2001). Past: paleontological statistics software package for education and data analysis. *Palaeontol. Electron.* 4:178.

[B32] Hara S. Morikawa T. Wasai S. Kasahara Y. Koshiba T. Yamazaki K. (2019). Identification of nitrogen-fixing bradyrhizobium associated with roots of field-grown sorghum by metagenome and proteome analyses. *Front. Microbiol.* 10:407. 10.3389/fmicb.2019.00407 30915047 PMC6422874

[B33] Hauggaard-Nielsen H. Mundus S. Jensen E. S. (2012). Grass-clover undersowing affects nitrogen dynamics in a grain legume-cereal arable cropping system. *F. Crop. Res*. 136, 23–31. 10.1016/j.fcr.2012.07.001

[B34] Herridge D. F. Peoples M. B. Boddey R. M. (2008). Global inputs of biological nitrogen fixation in agricultural systems. *Plant Soil* 311 1–18. 10.1007/s11104-008-9668-3

[B35] Horel Á Gelybó G. Potyó I. Pokovai K. Bakacsi Z. (2019). Soil nutrient dynamics and nitrogen fixation rate changes over plant growth in temperate soil. *Agronomy* 9:179 10.3390/agronomy9040179

[B36] Hsu S.-F. Buckley D. H. (2008). Evidence for the functional significance of diazotroph community structure in soil. *ISME J.* 31:124. 10.1038/ismej.2008.82 18769458

[B37] Iannetta P. P. M. Young M. Bachinger J. Bergkvist G. Doltra J. Lopez-Bellido R. J. (2016). A comparative nitrogen balance and productivity analysis of legume and non-legume supported cropping systems: the potential role of biological nitrogen fixation. *Front. Plant Sci.* 7:1700. 10.3389/fpls.2016.01700 27917178 PMC5116563

[B38] Izquierdo J. A. Nüsslein K. (2006). Distribution of extensive nifH gene diversity across physical soil microenvironments. *Microb. Ecol.* 51 441–452. 10.1007/s00248-006-9044-x 16645928

[B39] Jenkins B. D. Steward G. F. Short S. M. Ward B. B. Zehr J. P. (2004). Fingerprinting diazotroph communities in the Chesapeake Bay by using a DNA macroarray. *Appl. Environ. Microbiol.* 70 1767–1776. 10.1128/aem.70.3.1767-1776.2004 15006803 PMC368353

[B40] Jones D. L. Magthab E. A. Gleeson D. B. Hill P. W. Sánchez-Rodríguez A. R. Roberts P. (2018). Microbial competition for nitrogen and carbon is as intense in the subsoil as in the topsoil. *Soil Biol. Biochem.* 117 72–82. 10.1016/j.soilbio.2017.10.024

[B41] Jungblut A. D. Neilan B. A. (2010). *NifH* gene diversity and expression in a microbial mat community on the McMurdo Ice Shelf, Antarctica. *Antarct. Sci*. 22, 117–122. 10.1017/S0954102009990514

[B42] Kermah M. Franke A. C. Adjei-Nsiah S. Ahiabor B. D. K. Abaidoo R. C. Giller K. E. (2017). Maize-grain legume intercropping for enhanced resource use efficiency and crop productivity in the Guinea savanna of northern Ghana. *F. Crop. Res.* 213 38–50. 10.1016/J.FCR.2017.07.008 29104356 PMC5614088

[B43] Krzywinski M. Schein J. Birol I. Connors J. Gascoyne R. Horsman D. (2009). Circos: an information aesthetic for comparative genomics. *Genome Res.* 19 1639–1645. 10.1101/gr.092759.109 19541911 PMC2752132

[B44] Layek J. Das A. Mitran T. Nath C. Meena R. S. Yadav G. S. (2018). “Cereal+legume intercropping: an option for improving productivity and sustaining soil health,” in *Legumes for Soil Health and Sustainable Management*, eds Meena R. Das A. Yadav G. Lal R. (Singapore: Springer), 347–386. 10.1007/978-981-13-0253-4_11

[B45] Leite J. M. Ciampitti I. A. Mariano E. Vieira-Megda M. X. Trivelin P. C. O. (2016). Nutrient partitioning and stoichiometry in unburnt sugarcane ratoon at varying yield levels. *Front. Plant Sci*. 7:466. 10.3389/fpls.2016.00466 27148297 PMC4837160

[B46] Li C. Dong Y. Li H. Shen J. Zhang F. (2016a). Shift from complementarity to facilitation on P uptake by intercropped wheat neighboring with faba bean when available soil P is depleted. *Sci. Rep.* 6:18663. 10.1038/srep18663 26728339 PMC4700499

[B47] Li D. Voigt T. B. Kent A. D. (2016b). Plant and soil effects on bacterial communities associated with *Miscanthus* × *giganteus* rhizosphere and rhizomes. *GCB Bioenergy* 8 183–193. 10.1111/gcbb.12252

[B48] Li X. Sun M. Zhang H. Xu N. Sun G. (2016c). Use of mulberry-soybean intercropping in salt-alkali soil impacts the diversity of the soil bacterial community. *Microb. Biotechnol.* 9 293–304. 10.1111/1751-7915.12342 26892826 PMC4835568

[B49] Li Q. Chen J. Wu L. Luo X. Li N. Arafat Y. (2018). Belowground interactions impact the soil bacterial community, soil fertility, and crop yield in maize/peanut intercropping systems. *Int. J. Mol. Sci.* 19:622. 10.3390/ijms19020622 29470429 PMC5855844

[B50] Li X. Mu Y. Cheng Y. Liu X. Nian H. (2013). Effects of intercropping sugarcane and soybean on growth, rhizosphere soil microbes, nitrogen and phosphorus availability. *Acta Physiol. Plant.* 35 1113–1119. 10.1007/s11738-012-1148-y

[B51] Li X. Penttinen P. Gu Y. Zhang X. (2012). Diversity of nifH gene in rhizosphere and non-rhizosphere soil of tobacco in Panzhihua, China. *Ann. Microbiol.* 62 995–1001. 10.1007/s13213-011-0339-x

[B52] Li Y. R. Yang L. T. (2015). Sugarcane agriculture and sugar industry in China. *Sugar Tech* 17 1–8. 10.1007/s12355-014-0342-1

[B53] Lian T. Mu Y. Jin J. Ma Q. Cheng Y. Cai Z. (2019). Impact of intercropping on the coupling between soil microbial community structure, activity, and nutrient-use efficiencies. *PeerJ* 2019:e6412. 10.7717/peerj.6412 30775180 PMC6369829

[B54] Liu J. Peng M. Li Y. (2012). Phylogenetic diversity of nitrogen-fixing bacteria and the *nifH* gene from mangrove rhizosphere soil. *Can. J. Microbiol.* 58 531–539. 10.1139/w2012-016 22455729

[B55] Lovell C. R. Friez M. J. Longshore J. W. Bagwell C. E. (2001). Recovery and phylogenetic analysis of nifh sequences from diazotrophic bacteria associated with dead aboveground biomass of spartina alterniflora. *Appl. Environ. Microbiol.* 67 5308–5314. 10.1128/AEM.67.11.5308-5314.2001 11679360 PMC93305

[B56] Maidak B. L. Cole J. R. Lilburn T. G. Parker C. T. Jr. Saxman P. R. Farris R. J. (2001). The RDP-II (Ribosomal Database Project). *Nucleic Acids Res*. 29, 173–174. 10.1093/nar/29.1.173 11125082 PMC29785

[B57] Metsalu T. Vilo J. (2015). ClustVis: a web tool for visualizing clustering of multivariate data using Principal Component Analysis and heatmap. *Nucleic Acids Res.* 43 W566–W570. 10.1093/nar/gkv468 25969447 PMC4489295

[B58] Niederberger T. D. Sohm J. A. Tirindelli J. Gunderson T. Capone D. G. Carpenter E. J. (2012). Diverse and highly active diazotrophic assemblages inhabit ephemerally wetted soils of the Antarctic Dry Valleys. *FEMS Microbiol. Ecol*. 82, 376–390. 10.1111/j.1574-6941.2012.01390.x 22500944

[B59] Norman J. S. Friesen M. L. (2017). Complex N acquisition by soil diazotrophs: how the ability to release exoenzymes affects N fixation by terrestrial free-living diazotrophs. *ISME J.* 11 315–326. 10.1038/ismej.2016.127 27898052 PMC5270568

[B60] Nyoki D. Ndakidemi P. A. (2018a). Selected Chemical properties of soybean rhizosphere soil as influenced by cropping systems, rhizobzium inoculation, and the supply of phosphorus and potassium after two consecutive cropping seasons. *Int. J. Agron.* 2018:3426571 10.1155/2018/3426571

[B61] Nyoki D. Ndakidemi P. A. (2018b). Yield response of intercropped soybean and maize under rhizobia (Bradyrhizobium japonicum) Inoculation and P and K fertilization. *Commun. Soil Sci. Plant Anal.* 49 1168–1185. 10.1080/00103624.2018.1455846

[B62] Orr C. H. James A. Leifert C. Cooper J. M. Cummings S. P. (2011). Diversity and activity of free-living nitrogen-fixing bacteria and total bacteria in organic and conventionally managed soils. *Appl. Environ. Microbiol.* 77 911–919. 10.1128/AEM.01250-10 21131514 PMC3028727

[B63] Pankievicz V. C. S. do Amaral F. P. Santos K. F. D. N. Agtuca B. Xu Y. Schueller M. J. (2015). Robust biological nitrogen fixation in a model grass-bacterial association. *Plant J.* 81 907–919. 10.1111/tpj.12777 25645593

[B64] Paungfoo-Lonhienne C. Lonhienne T. G. A. Yeoh Y. K. Donose B. C. Webb R. I. Parsons J. (2016). Crosstalk between sugarcane and a plant-growth promoting Burkholderia species. *Sci. Rep.* 6:37389. 10.1038/srep37389 27869215 PMC5116747

[B65] Pérez-Montaño F. Alías-Villegas C. Bellogín R. A. Del Cerro P. Espuny M. R. Jiménez-Guerrero I. (2014). Plant growth promotion in cereal and leguminous agricultural important plants: from microorganism capacities to crop production. *Microbiol. Res.* 169 325–336. 10.1016/j.micres.2013.09.011 24144612

[B66] Pisa G. Magnani G. S. Weber H. Souza E. M. Faoro H. Monteiro R. A. (2011). Diversity of 16S rRNA genes from bacteria of sugarcane rhizosphere soil. *Brazilian J. Med. Biol. Res. Rev. Bras. Pesqui. Med. Biol.* 44 1215–1221. 10.1590/s0100-879x2011007500148 22042267

[B67] Plassart P. Terrat S. Thomson B. Griffiths R. Dequiedt S. Lelievre M. (2012). Evaluation of the ISO Standard 11063 DNA extraction procedure for assessing soil microbial abundance and community structure. *PLoS One* 7:44279. 10.1371/journal.pone.0044279 22984486 PMC3439486

[B68] Poly F. Ranjard L. Nazaret S. Gourbière F. Monrozier L. J. (2001). Comparison of nifH gene pools in soils and soil microenvironments with contrasting properties. *Appl. Environ. Microbiol.* 67 2255–2262. 10.1128/AEM.67.5.2255-2262.2001 11319109 PMC92864

[B69] Rascovan N. Carbonetto B. Perrig D. Díaz M. Canciani W. Abalo M. (2016). Integrated analysis of root microbiomes of soybean and wheat from agricultural fields. *Sci. Rep.* 6:28084. 10.1038/srep28084 27312589 PMC4911569

[B70] Rilling J. I. Acuña J. J. Sadowsky M. J. Jorquera M. A. (2018). Putative nitrogen-fixing bacteria associated with the rhizosphere and root endosphere of wheat plants grown in an andisol from southern chile. *Front. Microbiol.* 9:2710. 10.3389/fmicb.2018.02710 30524385 PMC6256256

[B71] Roley S. S. Xue C. Hamilton S. K. Tiedje J. M. Robertson G. P. (2019). Isotopic evidence for episodic nitrogen fixation in switchgrass (Panicum virgatum L.). *Soil Biol. Biochem.* 129 90–98. 10.1016/j.soilbio.2018.11.006

[B72] Rouws L. F. M. Leite J. de Matos G. F. Zilli J. E. Coelho M. R. R. Xavier G. R. (2014a). Endophytic Bradyrhizobium spp. isolates from sugarcane obtained through different culture strategies. *Environ. Microbiol. Rep.* 6 354–363. 24992534 10.1111/1758-2229.12122

[B73] Rouws L. F. M. Leite J. de Matos G. F. Zilli J. E. Coelho M. R. R. Xavier G. R. (2014b). Endophytic Bradyrhizobium spp. isolates from sugarcane obtained through different culture strategies. *Environ. Microbiol. Rep.* 6 354–363. 10.1111/1758-2229.12122 24992534

[B74] Shukla S. K. Yadav R. L. Singh P. N. Singh I. (2009). Potassium nutrition for improving stubble bud sprouting, dry matter partitioning, nutrient uptake and winter initiated sugarcane (*Saccharum* spp. hybrid complex) ratoon yield. *Eur. J. Agron*. 30, 27–33. 10.1016/j.eja.2008.06.005

[B75] Singh S. N. Yadav R. L. Yadav D. V. Singh P. R. Singh I. (2010). Introducing autumn Sugarcane as a relay intercrop in skipped row planted rice–potato cropping system for enhanced productivity and profitability in the Indian sub-tropics. *Exp. Agric.* 46 519–530. 10.1017/S001447971000058X

[B76] Solanki M. K. Wang F.-Y. Wang Z. Li C.-N. Lan T.-J. Singh R. K. (2019a). Rhizospheric and endospheric diazotrophs mediated soil fertility intensification in sugarcane-legume intercropping systems. *J. Soils Sediments* 19 1911–1927. 10.1007/s11368-018-2156-3

[B77] Solanki M. K. Wang F. Y. Li C. N. Wang Z. Lan T. J. Singh R. K. (2019b). Impact of sugarcane–legume intercropping on diazotrophic microbiome. *Sugar Tech*. 22 52–64. 10.1007/s12355-019-00755-4

[B78] Solanki M. K. Wang Z. Wang F. Y. Li C. N. Lan T. J. Singh R. K. (2017). Intercropping in Sugarcane cultivation influenced the soil properties and enhanced the diversity of vital diazotrophic bacteria. *Sugar Tech.* 19 136–147. 10.1007/s12355-016-0445-y

[B79] Szymańska S. Borruso L. Brusetti L. Hulisz P. Furtado B. Hrynkiewicz K. (2018). Bacterial microbiome of root-associated endophytes of Salicornia europaea in correspondence to different levels of salinity. *Environ. Sci. Pollut. Res.* 25 25420–25431. 10.1007/s11356-018-2530-0 29951760 PMC6133108

[B80] Tai X. S. Mao W. L. Liu G. X. Chen T. Zhang W. Wu X. K. (2013). High diversity of nitrogen-fixing bacteria in the upper reaches of the Heihe River, northwestern China. *Biogeosciences* 10 5589–5600. 10.5194/bg-10-5589-2013

[B81] Tai X. S. Mao W. L. Liu G. X. Chen T. Zhang W. Wu X. K. (2014). Distribution of ammonia oxidizers in relation to vegetation characteristics in the Qilian Mountains, northwestern China. *Biogeosci. Discuss*. 11, 5123–5146. 10.5194/bgd-11-5123-2014

[B82] Thomas R. J. Hungria M. (1988). Effect of potassium on nitrogen fixation, nitrogen transport, and nitrogen harvest index of bean. *J. Plant Nutr.* 11 175–188. 10.1080/01904168809363794

[B83] Thorburn P. J. Biggs J. S. Palmer J. Meier E. A. Verburg K. Skocaj D. M. (2017). Prioritizing crop management to increase nitrogen use efficiency in australian sugarcane crops. *Front. Plant Sci.* 8:1504. 10.3389/fpls.2017.01504 28928756 PMC5591824

[B84] Verma R. K. Yadav A. Rahman L. U. Kalra A. Patra D. D. (2014). Influence the status of soil chemical and biological properties by intercropping. *Int. J. Recycl. Org. Waste Agric.* 3 1–7. 10.1007/s40093-014-0046-2

[B85] Vitousek P. M. Cassman K. Cleveland C. Crews T. Field C. B. Grimm N. B. (2002). Towards an ecological understanding of biological nitrogen fixation. *Biogeochemistry* 57 1–45. 10.1023/A:1015798428743

[B86] Wang Z. G. Jin X. Bao X. G. Li X. F. Zhao J. H. Sun J. H. (2014). Intercropping enhances productivity and maintains the most soil fertility properties relative to sole cropping. *PLoS One* 9:e113984. 10.1371/journal.pone.0113984 25486249 PMC4259307

[B87] Wasai-Hara S. Hara S. Morikawa T. Sugawara M. Takami H. Yoneda J. (2020). Diversity of bradyrhizobium in non-leguminous sorghum plants: B. ottawaense isolates unique in genes for n2o reductase and lack of the type VI secretion system. *Microbes Environ.* 35 1–6. 10.1264/jsme2.ME19102 31932539 PMC7104290

[B88] Wedage W. M. M. Aberathne A. H. M. N. R. Harischandra I. N. Gunawardana D. (2019). A nodulation-proficient nonrhizobial inhabitant of *Pueraria phaseoloides*. *Sci. World J.* 2019:9782684. 10.1155/2019/9782684 31057340 PMC6463565

[B89] Yang W. Li Z. Wang J. Wu P. Zhang Y. (2013). Crop yield, nitrogen acquisition and sugarcane quality as affected by interspecific competition and nitrogen application. *F. Crop. Res.* 146 44–50. 10.1016/J.FCR.2013.03.008

[B90] Yousuf B. Kumar R. Mishra A. Jha B. (2014). Differential distribution and abundance of diazotrophic bacterial communities across different soil niches using a gene-targeted clone library approach. *FEMS Microbiol. Lett.* 360 117–125. 10.1111/1574-6968.12593 25196726

[B91] Zaeem M. Nadeem M. Pham T. H. Ashiq W. Ali W. Gilani S. S. M. (2019). The potential of corn-soybean intercropping to improve the soil health status and biomass production in cool climate boreal ecosystems. *Sci. Rep*. 9, 1–17. 10.1038/s41598-019-49558-3 31511594 PMC6739473

[B92] Zehr J. P. Jenkins B. D. Short S. M. Steward G. F. (2003). Nitrogenase gene diversity and microbial community structure: a cross-system comparison. *Environ. Microbiol.* 5 539–554. 10.1046/j.1462-2920.2003.00451.x 12823187

[B93] Zehr J. P. Mellon M. T. Zani S. (1998). New nitrogen-fixing microorganisms detected in oligotrophic oceans by amplification of Nitrogenase (nifH) genes. *Appl. Environ. Microbiol.* 64 3444–3450. 10.1128/aem.64.9.3444-3450.1998 9726895 PMC106745

[B94] Zhang F. Li L. (2003). Using competitive and facilitative interactions in intercropping systems enhances crop productivity and nutrient-use efficiency. *Plant Soil* 248 305–312. 10.1023/A:1022352229863

[B95] Zhang L.-H. Chen S.-F. (2012). Pseudacidovorax intermedius NH-1, a novel marine nitrogen-fixing bacterium isolated from the South China Sea. *World J. Microbiol. Biotechnol.* 28 2839–2847. 10.1007/s11274-012-1093-3 22806723

[B96] Zhang Y. Yang Q. Ling J. Van Nostrand J. D. Shi Z. Zhou J. (2017). Diversity and structure of diazotrophic communities in mangrove rhizosphere, revealed by high-throughput sequencing. *Front. Microbiol.* 8:2032. 10.3389/fmicb.2017.02032 29093705 PMC5651520

[B97] Zhou Y. Zhu H. Fu S. Yao Q. (2017). Variation in soil microbial community structure associated with different legume species is greater than that associated with different grass species. *Front. Microbiol.* 8:1007. 10.3389/fmicb.2017.01007 28620371 PMC5449475

[B98] Zong N. Jiang J. Shi P. Song M. Shen Z. Zhang X. (2015). Nutrient enrichment mediates the relationships of soil microbial respiration with climatic factors in an alpine meadow. *Sci. World J.* 2015:617471. 10.1155/2015/617471 26347902 PMC4549573

[B99] Zou Y. Zhang J. Yang D. Chen X. Zhao J. Xiu W. (2011). Effects of different land use patterns on nifH genetic diversity of soil nitrogen-fixing microbial communities in Leymus Chinensis steppe. *Acta Ecol. Sin.* 31 150–156. 10.1016/J.CHNAES.2011.03.004

