# Supplementary material for: Assessment of Diazotrophic Proteobacteria in Sugarcane Rhizosphere When Intercropped With Legumes (Peanut and Soybean) in the Field
Source: Front Microbiol. 2020 Jul 31;11:1814. doi: 10.3389/fmicb.2020.01814 (PMC7412970; doi:10.3389/fmicb.2020.01814)
Supplement: Supplementary file 1 [file Data_Sheet_1.docx]

**Supplementary material**

Table S1. Soil and weather details of both filed that used in this study.

| **Soil details*** | Field 1 | Field 2 |
| --- | --- | --- |
| pH | 5.85 | 5.17 |
| Soil organic carbon | 10.42g kg^-1^ | 26.45 g kg^-1^ |
| Total N | 0.94 g kg^-1^ | 1.45 g kg^-1^ |
| Total P | 0.53 g kg^-1^ | 1.08 g kg^-1^ |
| Total K | 8.99 g kg^-1^ | 4.97 g kg^-1^ |
| NH_4_^+^- N | 2.68 mg kg^-1^ | 9.05 mg kg^-1^ |
| NO_3_^-^N | 1.39 mg kg^-1^ | 4.39 mg kg^-1^ |
| Available P | 18.02 mg kg^-1^ | 33.36 mg kg^-1^ |
| Exchangeable K | 116.52 mg kg^-1^ | 70.24 mg kg^-1^ |
| **Weather** |  |  |
| Low temperature | 18.51±1.45 | 19.15±1.56 |
| High temperature | 26.05±1.52 | 26.34±1.62 |
| Precipitation | 98.21±19.94 | 101.14±20.44 |
| Before planting, the soil was fertilized with compound fertilizer (375 kg ha^-1^); contains fused calcium magnesium phosphate (75 kg ha^-1^: P_2_O_5_ 18%) and potassium chloride (150 kg ha^-1^: K_2_O 60%) and urea (150 kg ha^-1^: N 46%). | | |

*5 days before plantation

**Table S2 Details of Immumina MiSeq sequencing results of rhizospheric samples (**S only-Sugarcane monoculture, S+P-Sugarcane and Peanut intercropping, S+S-Sugarcane and Soybean intercropping)

| **Field no.** | **Growth stages** | **Cropping system** | **Sample code** | **Total Sequence** | **Filtered sequence** | **Total pairs read numbers** | **Non-Chimaric sequence** | **OTUs/ observed species** |
| --- | --- | --- | --- | --- | --- | --- | --- | --- |
| 1 | Tillering | S only | S1SM | 40074 | 39135 | 27912 | 12552 | 167 |
| 1 |  | S+P | S1SP | 39882 | 39057 | 26508 | 12553 | 182 |
| 1 |  | S+S | S1SS | 40570 | 39545 | 29537 | 17067 | 237 |
| 1 | Elongation | S only | S2SM | 40655 | 39355 | 32475 | 14347 | 243 |
| 1 |  | S+P | S2SP | 40976 | 39426 | 32160 | 14665 | 225 |
| 1 |  | S+S | S2SS | 40838 | 39364 | 30971 | 12825 | 210 |
| 1 | Maturation | S only | S3SM | 40878 | 39321 | 29395 | 15044 | 259 |
| 1 |  | S+P | S3SP | 41242 | 39924 | 31865 | 13488 | 224 |
| 1 |  | S+S | S3SS | 40835 | 39499 | 28861 | 13518 | 225 |
| 2 | Tillering | S only | D1SM | 39857 | 39017 | 34857 | 11306 | 114 |
| 2 |  | S+P | D1SP | 40795 | 39909 | 34997 | 14892 | 155 |
| 2 |  | S+S | D1SS | 40577 | 39751 | 34362 | 12878 | 151 |
| 2 | Elongation | S only | D2SM | 41003 | 39791 | 33982 | 12835 | 198 |
| 2 |  | S+P | D2SP | 40906 | 36870 | 33075 | 10628 | 166 |
| 2 |  | S+S | D2SS | 41199 | 39953 | 36485 | 10663 | 151 |
| 2 | Maturation | S only | D3SM | 40801 | 39533 | 34017 | 12799 | 208 |
| 2 |  | S+P | D3SP | 40589 | 39238 | 33640 | 12758 | 186 |
| 2 |  | S+S | D3SS | 40722 | 39531 | 35457 | 12024 | 174 |
| 1 | Before plantation | | S0 | 39895 | 39002 | 29203 | 15294 | 203 |
| 2 | Before plantation | | D0 | 39998 | 39062 | 34386 | 13131 | 140 |

**Table S3: Biosample IDs of samples that used in present study under the Bio-Project accession number PRJNA310619.**

| **Field no.** | **Growth stages** | **Cropping system** | **Sample code** | **Biosample ID** |
| --- | --- | --- | --- | --- |
| 1 | Tillering | S only | S1SM | SAMN04453312 |
| 1 |  | S+P | S1SP | SAMN04453313 |
| 1 |  | S+S | S1SS | SAMN04453314 |
| 1 | Elongation | S only | S2SM | SAMN04453315 |
| 1 |  | S+P | S2SP | SAMN04453316 |
| 1 |  | S+S | S2SS | SAMN04453317 |
| 1 | Maturation | S only | S3SM | SAMN04453318 |
| 1 |  | S+P | S3SP | SAMN04453319 |
| 1 |  | S+S | S3SS | SAMN04453320 |
| 2 | Tillering | S only | D1SM | SAMN04453295 |
| 2 |  | S+P | D1SP | SAMN04453296; |
| 2 |  | S+S | D1SS | SAMN04453297 |
| 2 | Elongation | S only | D2SM | SAMN04453298 |
| 2 |  | S+P | D2SP | SAMN04453299 |
| 2 |  | S+S | D2SS | SAMN04453300 |
| 2 | Maturation | S only | D3SM | SAMN04453301 |
| 2 |  | S+P | D3SP | SAMN04453302 |
| 2 |  | S+S | D3SS | SAMN04453303 |
| 1 | Before plantation | | S0 | SAMN04453311 |
| 2 | Before plantation | | D0 | SAMN04453294 |

**

**

**Figure S1:** Schematic diagram represents the cropping systems, sequencing, and analysis used in the present study.

S only-Sugarcane monoculture, S+P-Sugarcane and Peanut intercropping, S+S-Sugarcane and Soybean intercropping

**
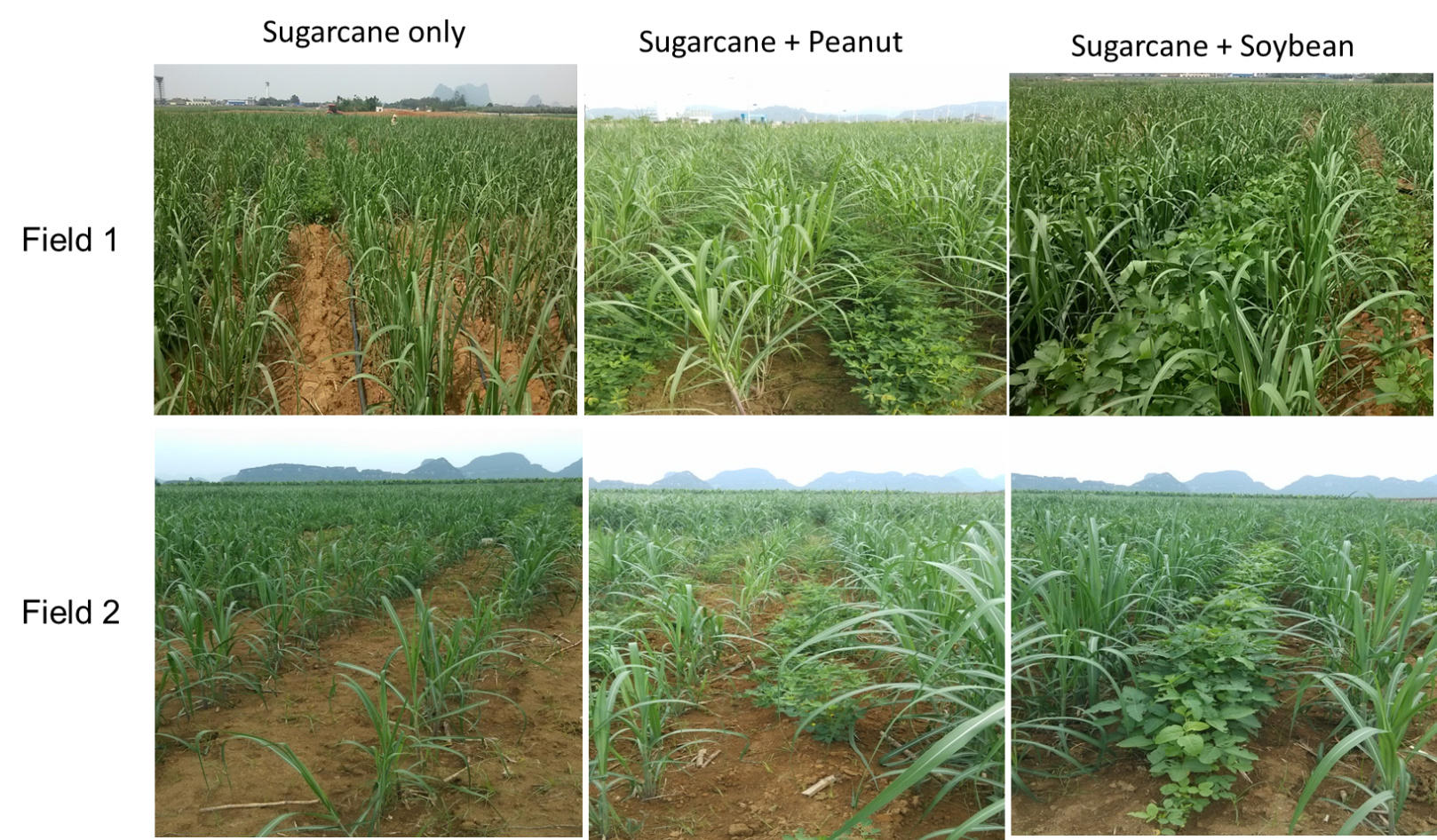
**

**Figure S2:** Sugarcane, peanut, and soybean intercropping in two different fields of Nanning, Guangxi, China.

**
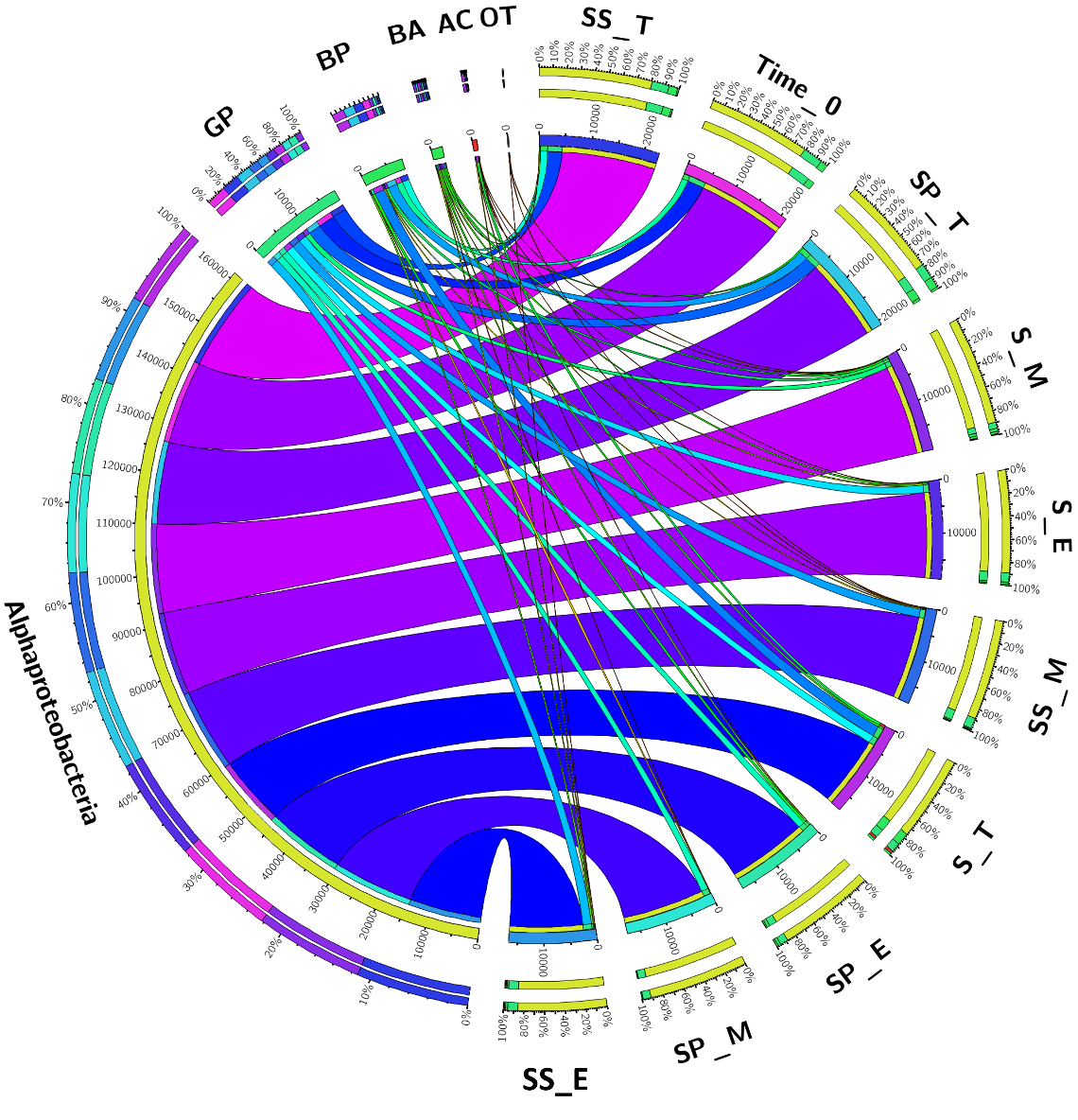
**

**Figure S3:** Circular representation of the proportional structure of bacterial communities at the class level, associated with the sugarcane rhizosphere in different growth stages. Taxa with a proportion lower than 0.1% in all samples are summarized as ‘OT-Others’. Values within the inner circle indicate the number of reads of a phylum and Order within the normalized dataset. S-Sugarcane only, SP-Sugarcane+Peanut, SS-Sugarcane+Soybean, T-Tillering, E-Elongation, M-Maturation, GP-*Gammaproteobacteria*, BP-*Betaproteobacteria*, BA-*Bacilli*, AC-*Actinobacteria*, OT-Others

**
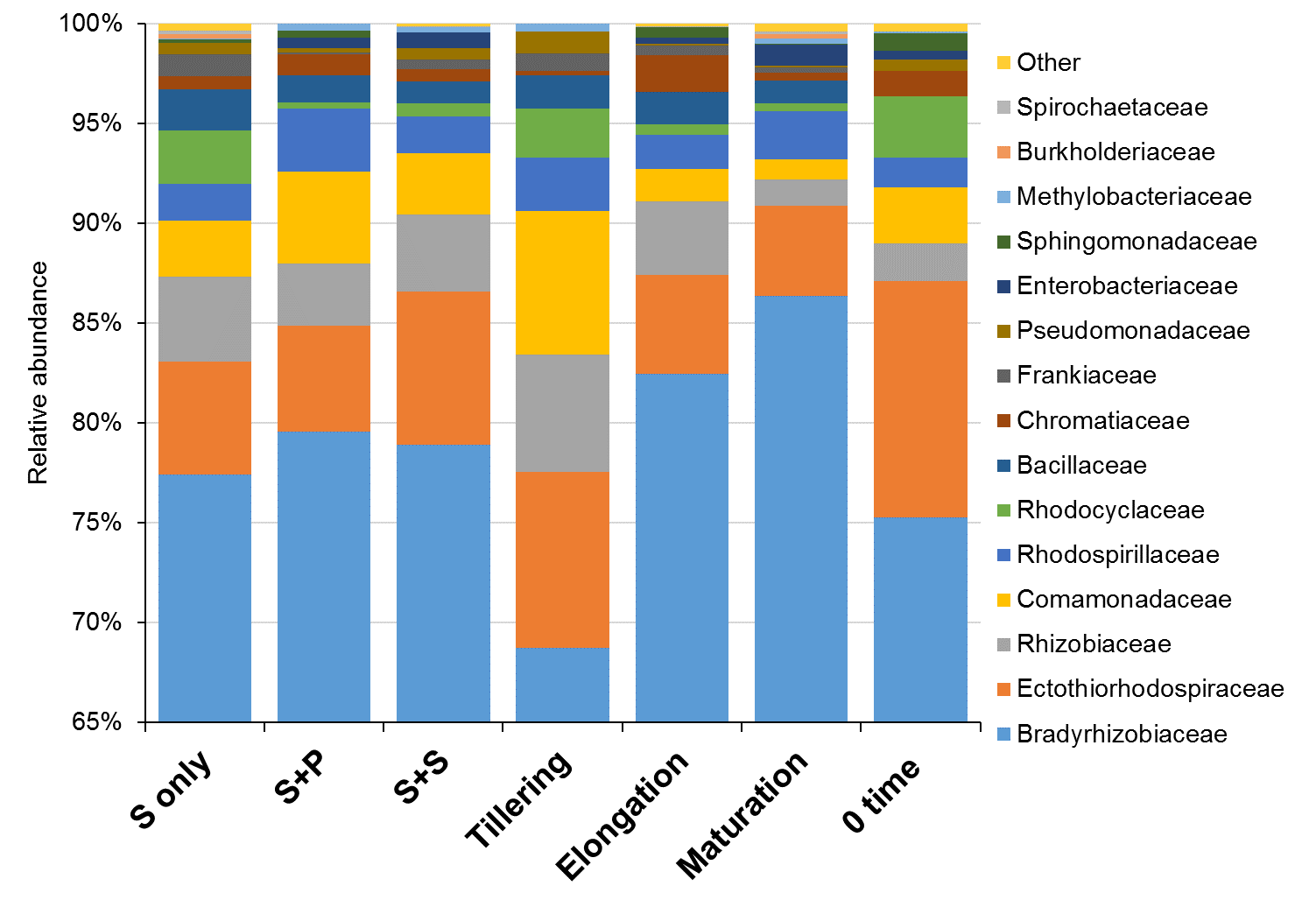
**

**Figure S4. Bar graph of relative abundance of diazotrophs at the family level.** The most abundant classes are shown. Taxa with a proportion lower than 0.1% in all samples are summarized as ‘Others’. S only-Sugarcane monoculture, S+P-Sugarcane and Peanut intercropping, S+S-Sugarcane and Soybean intercropping

**

**

**Figure S5.** Box plots showing microbial shift between cropping systems and growth stages. *P<0.05 (ANOVA by LSD test). S only-Sugarcane monoculture, S+P-Sugarcane and Peanut intercropping, S+S-Sugarcane and Soybean intercropping





**Figure S6.** Box plots showing sugarcane hight and yield. S only-Sugarcane monoculture, S+P-Sugarcane and Peanut intercropping, S+S-Sugarcane and Soybean intercropping
